# Structural Basis of PP2A Inhibition by Small t Antigen

**DOI:** 10.1371/journal.pbio.0050202

**Published:** 2007-07-03

**Authors:** Uhn Soo Cho, Seamus Morrone, Anna A Sablina, Jason D Arroyo, William C Hahn, Wenqing Xu

**Affiliations:** 1 Department of Biological Structure, University of Washington, Seattle, Washington, United States of America; 2 Department of Medical Oncology, Dana-Farber Cancer Institute, Harvard Medical School, Boston, Massachusetts, United States of America; 3 Broad Institute of Harvard and MIT, Cambridge, Massachusetts, United States of America; University of Wisconsin-Madison, United States of America

## Abstract

The SV40 small t antigen (ST) is a potent oncoprotein that perturbs the function of protein phosphatase 2A (PP2A). ST directly interacts with the PP2A scaffolding A subunit and alters PP2A activity by displacing regulatory B subunits from the A subunit. We have determined the crystal structure of full-length ST in complex with PP2A A subunit at 3.1 Å resolution. ST consists of an N-terminal J domain and a C-terminal unique domain that contains two zinc-binding motifs. Both the J domain and second zinc-binding motif interact with the intra-HEAT-repeat loops of HEAT repeats 3–7 of the A subunit, which overlaps with the binding site of the PP2A B56 subunit. Intriguingly, the first zinc-binding motif is in a position that may allow it to directly interact with and inhibit the phosphatase activity of the PP2A catalytic C subunit. These observations provide a structural basis for understanding the oncogenic functions of ST.

## Author Summary

The study of how DNA tumor viruses induce malignant transformation has led to the identification of key pathways that also play a role in spontaneously arising cancers. One such virus, simian virus 40 (SV40), produces two proteins, the large T and small t antigens, that bind and inactivate tumor suppressor genes important for cell transformation. Specifically, SV40 small t antigen (ST) binds to and perturbs the function of the abundant protein phosphatase 2A (PP2A). PP2A is a family of heterotrimeric enzymes, composed of a structural A subunit, a catalytic C subunit, and one of several regulatory B subunits. Here we have determined the structure of SV40 ST in complex with the PP2A structural subunit Aα. SV40 ST consists of an N-terminal J domain and a C-terminal unique domain that contains two separate zinc-binding motifs. SV40 ST binds to the same region of PP2A as the regulatory subunit B56, which provides a structural explanation for the displacement of regulatory B subunits by SV40 ST. Taken together, these observations provide a structural basis for understanding the oncogenic functions of ST.

## Abstract 3

The crystal structure of full-length SV40 small t antigen (ST) in complex with the A subunit of its target, protein phosphatase 2A, contributes to our understanding of the oncogenic functions of ST.

## Introduction

Simian virus 40 (SV40) is a DNA tumor virus in the polyomavirus family. SV40 may play a role in a subset of human cancers, and the study of transformation induced by SV40 has led to many insights into the pathways involved in spontaneously arising cancers [1]. The Early Region of SV40 is essential for transformation and encodes two oncoproteins, the large T antigen (LT) and small t antigen (ST), through alternative splicing. LT binds to a number of host proteins including the retinoblastoma and p53 tumor suppressors. ST, which shares its N terminus with LT but has a unique C-terminal end, is also a potent oncoprotein that plays a critical role in the transformation of several human cell types [2,3]. For example, the cointroduction of LT, ST, the telomerase catalytic subunit hTERT (human telomerase reverse transcriptase), and an oncogenic allele of H*-*RAS imparts a tumorigenic phenotype to a wide range of primary human cells [4–6]. The tumorigenic activity of ST is strictly dependent on its interaction with protein phosphatase 2A (PP2A), since mutant versions of ST that are unable to bind PP2A fail to induce tumorigenic activity [5].

PP2A is a large family of heterotrimeric enzymes that accounts for the majority of total Ser/Thr phosphatase activity in most tissues and cells [7–10]. Although a dimer comprised of a ~65 kDa scaffolding A subunit and a ~36 kDa catalytic C subunit constitutes the core enzymatic activity of PP2A, the binding of a third regulatory B subunit to the AC core enzyme regulates PP2A activity, cellular localization, and substrate specificity. PP2A can exist in cells as either the AC core complex or an ABC heterotrimeric holoenzyme that is the dominant form in most cells. The scaffold A subunit is composed of 15 HEAT (huntingtin, elongation factor 3, A subunit of protein phosphatase 2A, and target of rapamycin) repeats [11], while the C subunit contains a catalytic domain that shares sequence homology with other Ser/Thr phosphatases such as protein phosphatase 1 and protein phosphatase 2B (calcineurin). In humans there are at least 18 B subunits that can be classified into B (B55), B′ (B56), B′′, and B′′′ families based on sequence similarity.

PP2A regulates a wide array of cellular processes. In particular, several lines of evidence implicate PP2A as a tumor suppressor gene. Specifically, PP2A inhibitors such as microcystin and okadaic acid are potent tumor promoters and the ST antigen acts as a potent oncogene [12–16]. Consistent with its role as a tumor suppressor, low frequency mutations of the Aα and Aβ subunits are found in breast, lung, and colorectal cancers [12,17–20]. Mutations in PP2A Aα result in functional haploinsufficiency that depletes complexes containing B56γ, thereby leading to cell transformation [16,21]. In contrast, mutations in Aβ disrupt the interaction of Aβ with the small GTPase RalA, leading to constitutive RalA phosphorylation and activity [22].

ST interacts with the PP2A AC core dimer through the direct interaction between ST and the PP2A scaffolding A subunit [23,24]. Prior work has suggested that ST associates with the PP2A C subunit through interactions with the A subunit. While the C subunit interacts with HEAT repeats 11–15 of A subunit, ST interacts with HEAT repeats 3–7, which is also the binding site for PP2A B subunits [25,26]. The ability of ST to displace multiple B subunits from the A subunit has been demonstrated both in vitro [27,28] and in vivo [16,29]. The displacement of B subunits by ST inhibits PP2A activity towards multiple substrates, but increases phosphatase activity towards histone H1 [22,30]. Therefore, ST can be considered as a viral B subunit that changes the biochemical properties of PP2A.

The 174 amino acid ST shares its N-terminal 78 residues with the N-terminal sequence of LT, including a region that exhibits J domain (also termed the DnaJ domain) function [3,31,32]. The unique C-terminal 96 residues of ST antigen interact with the PP2A A subunit. The C-terminal unique domain is rich in Cys residues, six of which are organized into two CxCxxC clusters and bind two zinc ions, providing structural stability to ST [33,34]. The N-terminal J domain is not required for the interaction of ST with the A subunit of PP2A but may contribute to the high affinity binding, since the removal of the first 51 residues from ST caused a 140-fold reduction in inhibition towards the phosphatase activity of PP2A AC core complex [35]. The N-terminal J domain shares sequence homology with the DnaJ family of molecular co-chaperones, which promote the ATPase and chaperone activities of heat shock protein 70 (Hsp70), an important chaperone in the cell [36,37]. Hsp70 binding region has been mapped to the surface formed by J domain helices 2–3 [38,39]. The DnaJ-like domain in ST and the DnaJ domain in E. coli are interchangable without a loss in co-chaperone activity [40]. While the structure of the J domain can be predicted from prior structural determinations of LT, the overall structure of ST remains to be unraveled and it is unclear how ST may interact with PP2A and regulate PP2A activities.

We have determined the crystal structure of full-length SV40 ST in complex with the full-length Aα subunit of PP2A. This structure reveals two novel zinc-binding motifs formed by the unique C-terminal domain, the structural linkage of the J and unique domain of ST, and the interaction site of ST with the structural A subunit. Together with our biochemical data, we provide a structural basis for understanding the tumorigenic activity of ST protein.

## Results

### Overall Structure

**Figure 1:**
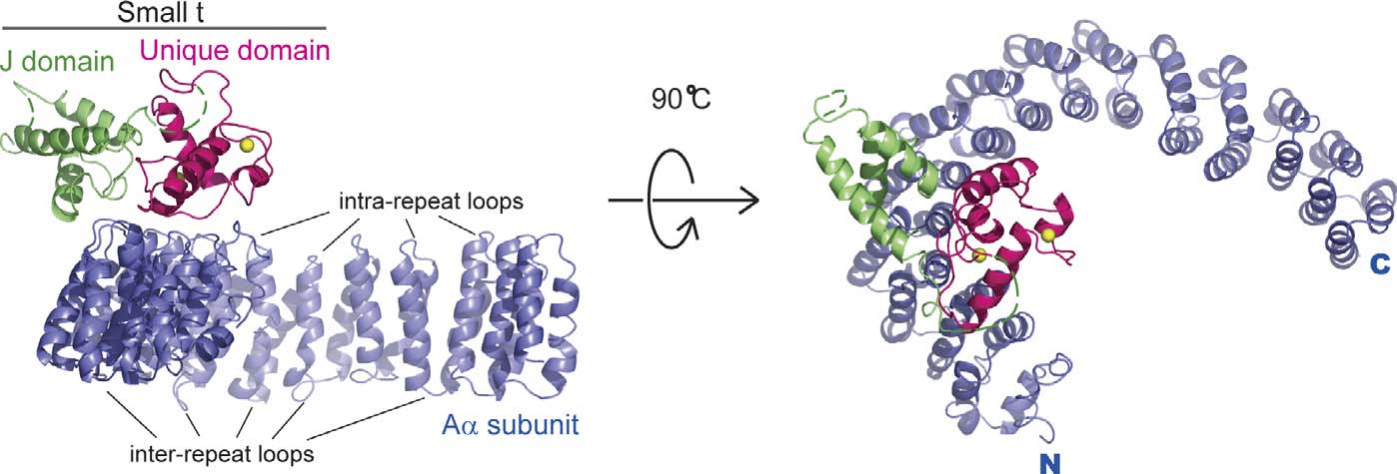
Overall Structure of SV40 ST in Complex with the A Subunit of PP2A A cartoon illustration of the “front” and “top” views of the PP2A A subunit–SV40 ST complex. The scaffold Aα subunit of PP2A, the J domain, and the unique domain of SV40 ST are colored blue, green, and magenta, respectively. In addition, two zinc ions in the ST unique domain are yellow. ST interacts with the intrarepeat loops of HEAT repeats 3–7 of PP2A A subunit.

The protein complex containing full-length SV40 ST and full length murine PP2A Aα subunit (A-ST complex) were co-expressed in E. coli and purified to homogeneity. Crystal structure of the complex was determined by a combination of molecular replacement, using the PP2A A subunit structure as the searching model, and single-wavelength anomalous dispersion of intrinsic zinc atoms in ST, and was refined at 3.1 Å resolution (Table 1). Four complexes were found in each asymmetric unit. In each complex, the scaffolding A subunit contains 15 HEAT repeats that forms a horseshoe shape. The four A-ST complexes in the asymmetric unit have essentially the same structure, except HEAT repeats 11–15 that show substantial conformational variation (see below). ST contains an N-terminal J domain and a C-terminal unique domain. These two domains sit on the concave and convex sides of the ridge of the A subunit horseshoe structure, respectively, by interacting with intra-repeat loops of the Aα subunit HEAT repeats 3–7 (Figure 1), which is also the binding site for B56γ1 in the A-B56γ1-C trimeric PP2A holoenzyme structure [25,26].

**Table 1:**
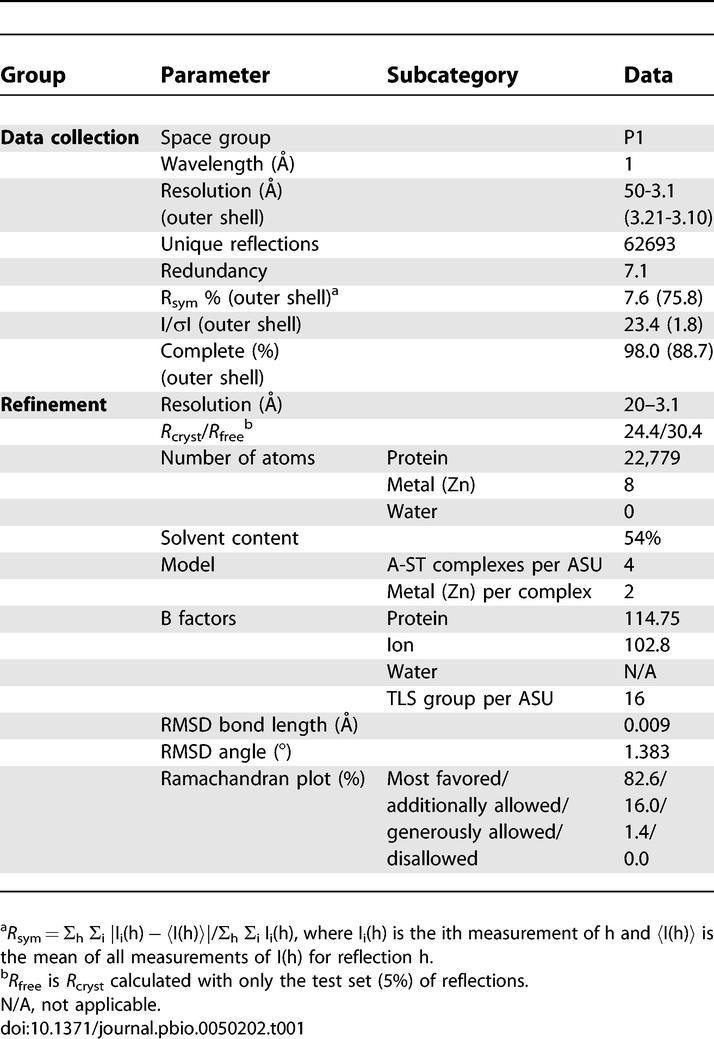
Summary of Crystallographic Analysis of the PP2A Aα-SV40 Small t Antigen Complex

### Structure of SV40 ST

**Figure 2:**
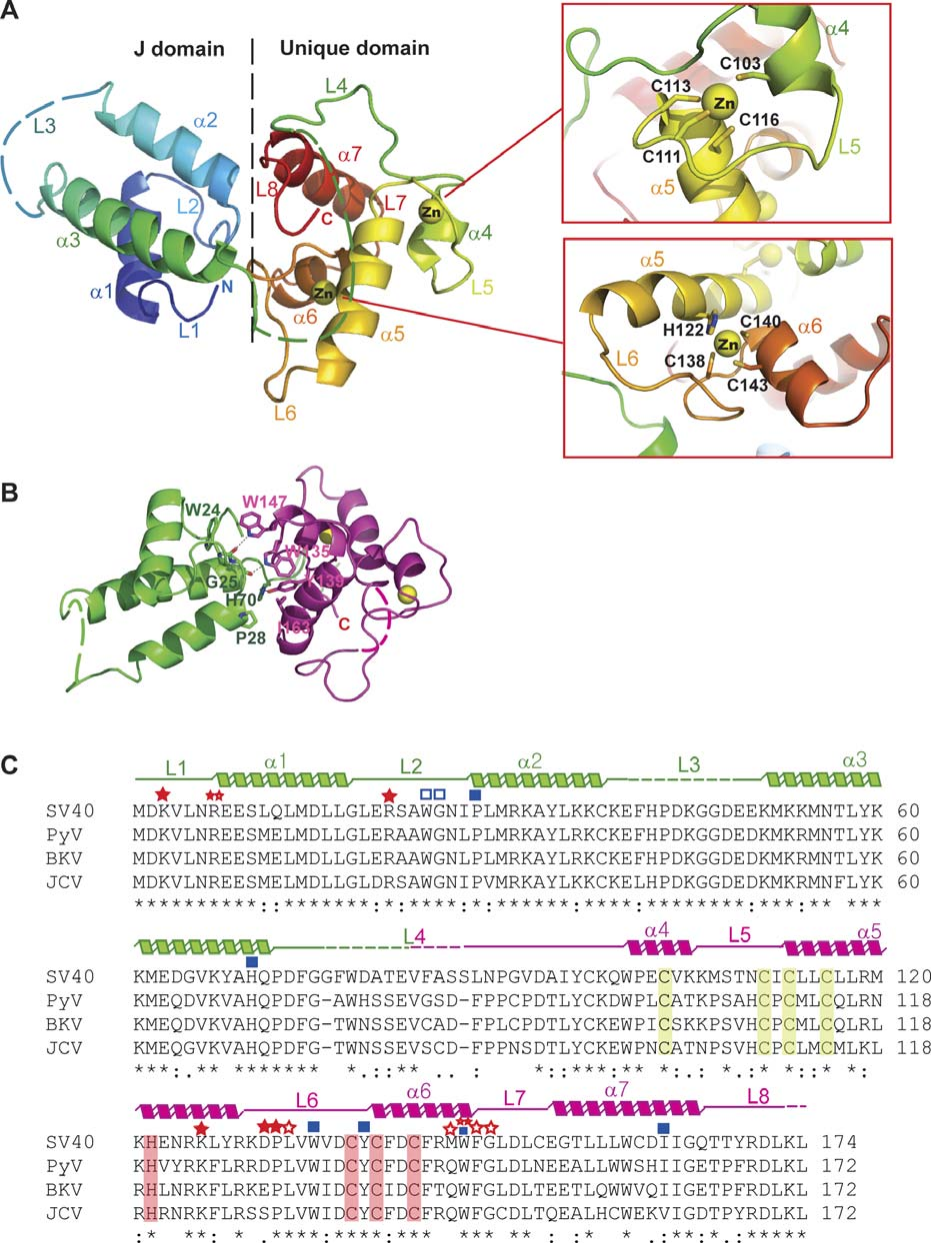
Structure of SV40 Small t Antigen (A) Structural organization of SV40 ST and the zinc coordination of ST unique domain. The peptide chain is color-coded from blue to red, going through the rainbow colors, from the N terminus (blue) to the C terminus (red). (B) Interface of the J and unique domains. The J and unique domains are in green and pink, respectively. Key residues in the interface between the J domain and the unique domain—Trp24, Gly25, Pro28, His70, Trp135, Tyr139, Trp147, and Ile163—are labeled. (C) Sequence alignment of ST proteins from SV40 (strain VA45-54-2), baboon polyomavirus (PyV), BK polyomavirus (BKV), and JC polyomavirus (JCV). Sequence conservation is indicated below the aligned sequences. Secondary structures in the determined crystal structure are indicated above the aligned sequences. Solid and empty stars indicate residues interacting with PP2A A subunit using side-chain and main-chain, respectively. Solid and empty squares represent residues involved in interactions between the J domain and the unique domain with side-chain and main-chain atoms, respectively.

SV40 ST exhibits an all α-helix structure with two zinc-binding sites in the unique domain (Figure 2A). The J domain contains three helices and has a structure similar to the previously solved crystal structure of the J domain of SV40 LT and a NMR structure of polyomavirus DnaJ-like domain [41,42], with Cα root mean square deviations (RMSDs) of 1.84 and 1.83 Å, respectively. The DnaJ domain in the E. coli DnaJ protein forms a complex with Hsp70 in the flexible L3 region [38,39]. Interestingly, the expected Hsp70 binding region of the J domain is not involved in the interaction with PP2A A subunit and appears widely accessible to Hsp70. In contrast, the Hsp70 binding region of J domain in LT is involved in the binding of retinoblastoma protein [41]. The linker between helices 2 and 3, which has been implicated in Hsp70 interaction, as well as the linker between J and unique domains (residues 72–86), is not visible in any of the four complexes in the asymmetric unit, suggesting structural flexibility of these two linkers in the A-ST complex.

The ST unique domain is composed of four helices. Three of these helices are directly involved in the interaction with two zinc ions. The first zinc-binding site is positioned between two roughly parallel helices (α4 and α5) and the L5 loop, coordinated by four conserved cysteine residues (Cys103, Cys111, Cys113, and Cys116). Cys103 stems from the C-terminal end of α4, while Cys113 and Cys116 are positioned in the N-terminal end of α5 (Figure 2A). The second zinc is located between two roughly perpendicular helices (α5 and α6) and coordinated by three conserved cysteines in the second cysteine cluster (Cys138, Cys140, and Cys143), with Cys140 and Cys143 from the N-terminal end of α6 and the conserved His122 from the middle of α5 (Figure 2A). Although these two zinc ions are located in discrete positions, both of them coordinate with α5. These two zinc-binding motifs are completely distinct from the previously proposed GAL4-type zinc cluster for ST and are also different from classical zinc finger structures [33,34,43].

The J and unique domains have an interface that is mostly hydrophobic (Figure 2B). These two domains of SV40 ST interact with each other by several hydrogen-bonding and hydrophobic interactions. The indole rings of Trp135 and Trp147 make hydrogen bonds with the backbone carbonyl group of Gly25 and Trp24, respectively. The hydroxyl group of Tyr139 forms a hydrogen bond with the imidazole ring of His70. Pro28 and Ile163 make a hydrophobic interaction between two domains (Figure 2B). It should be noted that both the N and C termini of ST are located at the interface of the J and unique domains . In particular, the N terminus is located in the joint point of J domain and the A subunit, and directly interacts with the A subunit. The interface and relative orientation of these two domains are essentially identical in all four A-ST complexes in the asymmetric unit. The Cα RMSDs of ST among these four complexes are between 0.95 Å and 1.03 Å. Therefore, the J and unique domains of ST are structurally coupled and ST consists of one globular fold. Since residues in the A-ST interface are highly conserved among ST proteins (Figure 2C), this domain organization should be conserved among ST proteins in the polyomavirus family.

### The Interface between ST and PP2A A Subunit

**Figure 3:**
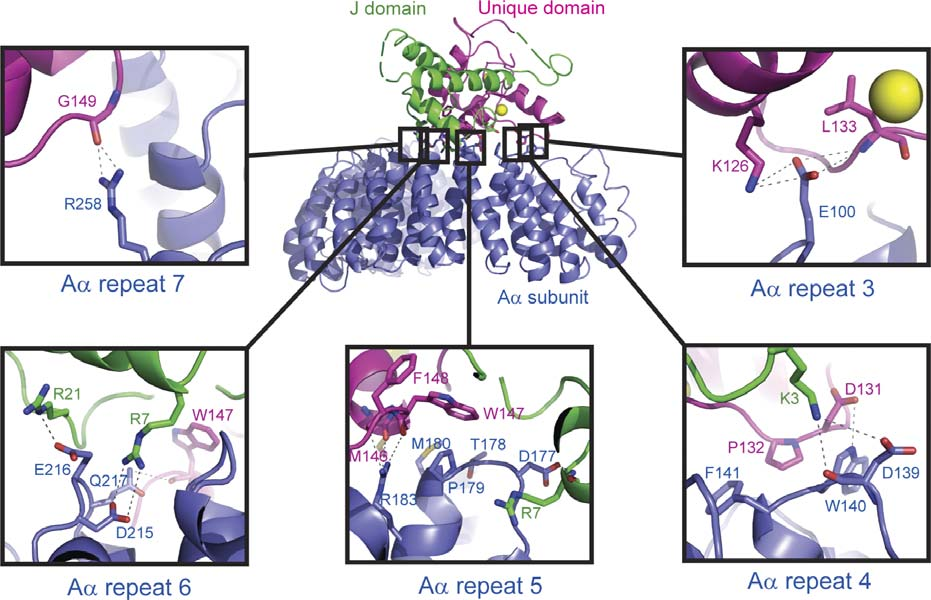
The Interface between SV40 ST and PP2A A Subunit Color assignment for different subunit or domains is the same as in Figure 1. Hydrogen bonds between ST and the PP2A A subunit are indicated by dashed lines.

The interactions between the Aα subunit and SV40 ST are formed through the intra-loop regions of repeats 3 to 7 in the Aα subunit (Figures 1 and 3). Detailed interactions are summarized in Figure 3. ST uses both its J domain and the second zinc-binding motif (in particular the L6 loop) for interacting with PP2A Aα subunit, while the first zinc finger is not directly involved in interactions with PP2A Aα subunit, but may be involved in interactions with PP2A catalytic C subunit (see below). One notable feature of this interaction is that five of eight residues in the ST unique domain (Figures 2C and 3) in the A-ST interface interact with the Aα subunit through the backbone of those residues. The backbone conformations of those ST residues (Leu133, Met146, Trp147, Phe148, and Gly149) are largely determined by the second zinc-binding site, demonstrating the importance of the second zinc-binding site in the Aα subunit interaction. In contrast, all of the Aα subunit residues interact with ST through their side chains, and some of those residues were identified by previous A subunit mutagenesis studies [44].

### Structural Comparison of A-ST Complex with A-B56-C PP2A Holoenzyme

**Figure 4:**
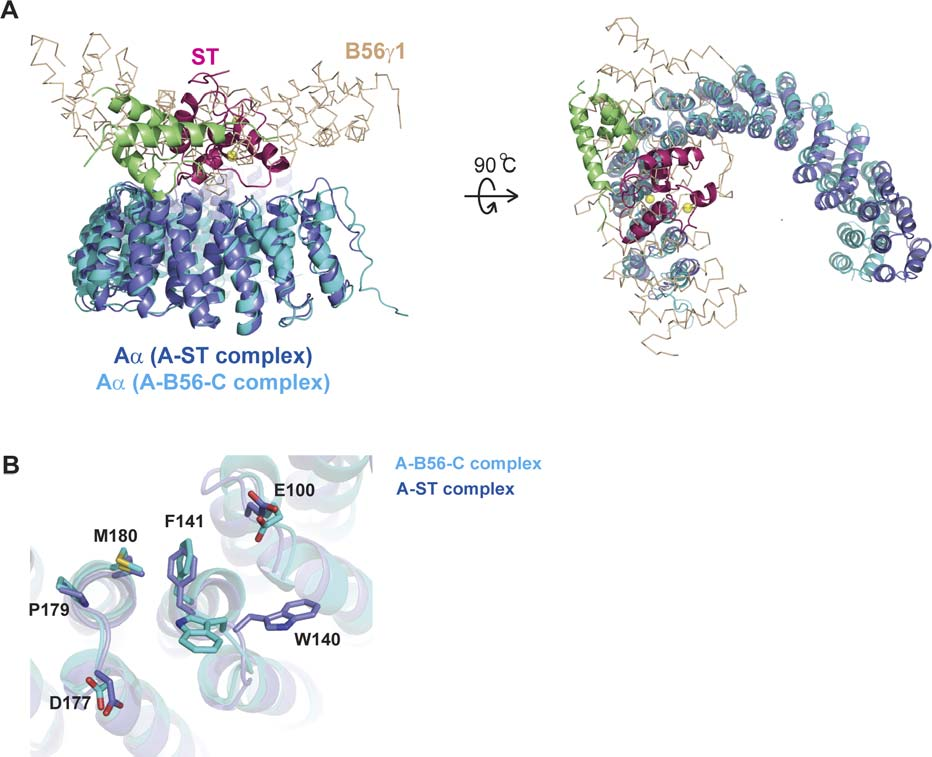
Structural Comparison of the A-ST Complex and the A-B56-C PP2A Holoenzyme (A) Structural superposition of these two complexes. These two complexes are superimposed using A subunit HEAT repeats 2–10. The J and unique domain of ST are colored green and pink, respectively. The Cα trace of B56γ1 are shown in light orange. It is clear that ST and B56γ1 bind to the same sites on PP2A A subunit. (B) The PP2A A subunit residues involved in both ST and B56γ1 interactions. ST and B56γ1 share a common footprint on the ridge of A subunit.

When the A-ST complex structure is superimposed with the previously reported A-B56-C PP2A holoenzyme structure [25,26], it is obvious that ST and the B56 subunit interact with the same region of PP2A A subunit and have very similar “footprints” on the scaffold A subunit (Figure 4A). The majority of Aα residues involved in the A-ST interaction—including Glu100, Trp140, Phe141, Asp177, Pro179, and Met180—are also involved in the A-B56 interaction (Figure 4B). The side chain conformations of those Aα residues are quite similar in both structures, except Trp140. The position of the indole ring of Trp140 is flipped between these two complex structures, mostly due to the intercalation of Pro132 of ST between Trp140 and Phe141 by forming a hydrophobic core (Figures 3 and 4B). The largely overlapping Aα binding sites of ST and B56 explains how ST competes with B56 for the binding of the Aα subunit [25,26].

**Figure 5:**
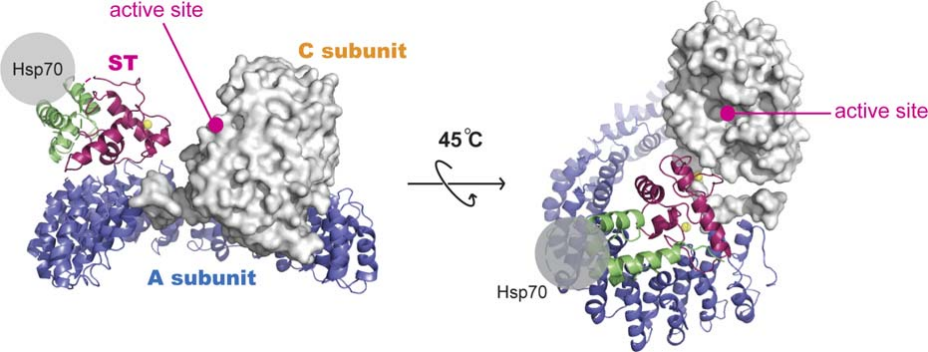
The First Zinc-Binding Motif May Directly Interact with and Inhibit the Catalytic C Subunit of PP2A The structures of the A-ST and A-B56-C complexes are superimposed. The PP2A catalytic subunit is shown in the surface model. The active site of the PP2A C subunit is indicated. The potential binding site of Hsp70 is represented by a gray sphere.

In the A-B56-C PP2A holoenzyme structure, both B56 and C subunits sit on the same side (intra-repeat loop side) of the horseshoe shape formed by the scaffolding A subunit [25,26]. When HEAT repeats 2–10 in the A-ST complex are superimposed with corresponding regions of the A-B56-C complex, the first zinc-binding motif, in particular helix α4 and its flanking region, is in close proximity to the C subunit active site (Figure 5). The closest atoms between ST and C subunit in this superposition are within van der Waals distances. It should be noted that, when the A-ST complex structure is superimposed with the PP2A AC core complex, the distance between the ST first zinc-binding motif and C subunit appears further apart than in the A-B56-C holoenzyme. However, since the PP2A A subunit has substantial structural flexibility (see below), it is plausible that ST may interact directly with the C subunit near its catalytic active site, just like the B56 subunit that interacts with both A and C subunits.

### Structural Comparison of the A Subunit Conformations in Different Complexes

**Figure 6:**
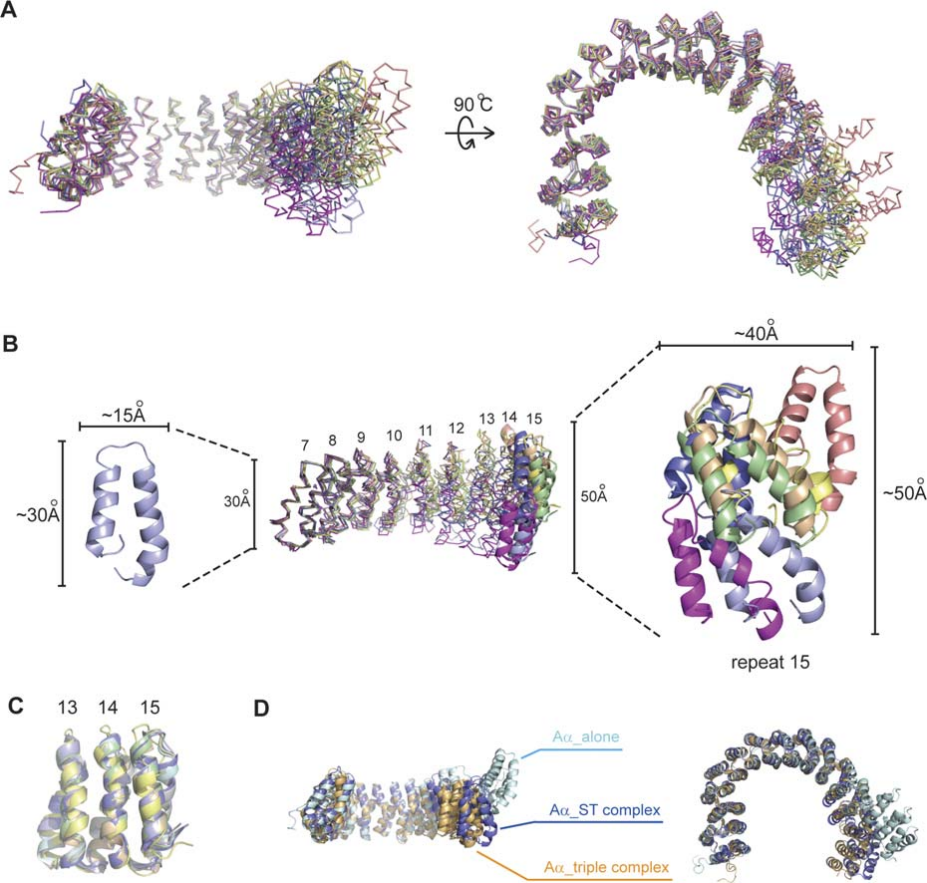
Structural Comparison of the PP2A A Subunit and the Structural Flexibility (A) The structural alignment of PP2A A subunits. PP2A A subunit structures were aligned based on their structures of HEAT repeats 2–10. PP2A A subunit structures that used for the alignment are from the four A-ST complexes in the asymmetric unit, the A subunit structure alone (PDB code: 1B3U), the AC dimer structure (PDB code: 2IE3), and the A-B56-C trimeric structure (PDB code: 2IAE). The HEAT repeats 2–10 may form a rigid structural block since no significant structural variations were observed for this region among all A subunit structures. (B) The amplitude of conformational variations of the PP2A A subunit HEAT repeats 11–15. (C) HEAT repeats 13–15 may form the other relatively rigid structural block in the PP2A A subunit. There is no major conformational variation in HEAT repeats 13–15 among all A subunit structures. Therefore, the structural variations observed in (A) and (B) are mostly due to the conformational flexibility of HEAT repeats 10–13. (D) Superposition of PP2A A subunit structures from Aα alone, A-ST complex, and A-B56-C trimeric complex. The structure of the PP2A A subunit in the A-ST complex can have a conformation very similar with that of the A subunit in the A-B56-C trimeric complex.

The A-ST interface and the structure of HEAT repeats 2–10 of the A subunit are quite rigid, since these structures can be well superimposed in all four A-ST complexes in the asymmetric unit. In contrast, HEAT repeats 11–15 apparently have highly variable structures, and the first HEAT repeat is not well folded in some cases (Figure 6A and 6B). While the RMSDs of all 15 HEAT repeats among four A-ST complexes in the asymmetric unit range from 1.29 Å to 3.02 Å, the RMSD of HEAT repeats 2–10 are on average 1.09 Å. The conformational variation of the Aα subunit results mostly from conformational flexibility in HEAT repeats 10–13, since HEAT repeats 13–15 among the four complexes are easily superimposed, with an average RMSD of 1.15 Å (Figure 6C). Structural comparison of the A-ST crystal structure with previously reported crystal structures of A, AC complex, and A-B56-C complexes [25,26,45,46] also support the conclusion that HEAT repeats 2–10 and HEAT repeats 13–15 form two relatively rigid blocks. However, there is substantial structural flexibility between these two structural blocks, due to the result of accumulative conformational changes in HEAT repeats 10–13 (Figures S1 and S2).

### Mutagenesis of the A-ST Interface

**Figure 7:**
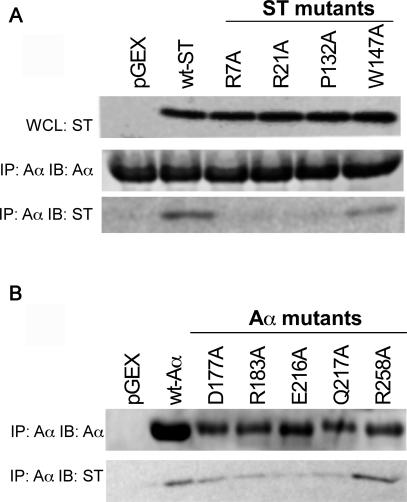
Mutational Analysis of the A-ST Interface (A) Mutation of ST residues predicted to interact with PP2A Aα. Top: Expression of ST in whole cell lysates (WCL). Middle: Isolation of PP2A Aα complexes and immunoblotting with anti-PP2A Aα antibodies. Bottom: Isolation of PP2A Aα complexes and immunoblotting for ST. (B) Mutation of PP2A Aα residues predicted to interact with ST. Top: Isolation of PP2A Aα complexes and immunoblotting with anti-PP2A Aα. Bottom: Isolation of PP2A Aα complexes and immunoblotting for ST.

To determine whether the amino acid residues observed in our structure to be interaction points between ST and Aα, we generated a set of ST (R7A, R21A, P132A, and W147A) and Aα (D177A, R183A, E216A, Q217A, and R258A) mutants and performed in vitro binding assays. We found that substitution of alanine for residues Arg7, Arg21, or Pro132 of ST abrogated interaction between ST and wild-type Aα (Figure 7). In addition, we found that the W147A ST mutant showed reduced binding to Aα compared to wild-type ST. Analysis of Aα mutants indicated that single alanine substitutions at position Glu216 disrupted PP2A A-ST interaction (Figure 7). These observations provide strong support to the A-ST interface observed in our crystal structure.

## Discussion

### Structure of Small t Antigen and Its Interaction with PP2A A Subunit

Here we report the crystal structure of SV40 ST in complex with the murine PP2A A subunit. It is striking that all four A-ST complexes in the asymmetric unit of our crystal lattice have essentially the same structure, except the flexible HEAT repeats 11–15 of the A subunit that are not involved in the A-ST interaction. This observation argues strongly that the ST structure as well as the A-ST interface observed in our crystal structure are independent of crystal packing and should be physiologically relevant. Since the human and murine Aα subunits are identical except for one residue that is distant from the ST binding site (Ser324 in human, Thr324 in mouse), this structure is likely representative of the interaction of ST with the human PP2A. Moreover, since most ST residues in the structural core and the A-ST interface are conserved among ST and middle t (MT) proteins in the polyomavirus family, this structure provides a structural basis for understanding the oncogenic activities of each of the ST and MT proteins in the polyomavirus family.

We found that the N-terminal J and the C-terminal unique domains of ST are structurally coupled. The conserved hydrophobic interface between the J and unique domains may be important for the structural integrity of ST*.* Previous studies have been shown that each ST molecule contains two zinc ions [33,34]. Since ST contains two cysteine cluster motifs (CXCXXC) that are absolutely conserved, it was proposed that ST may resemble GAL4, which contains a Zn(II)_2_Cys_6_ binuclear cluster [33,34]. In our ST crystal structure, instead of forming a binuclear cluster, these two zinc ions are located in two separate positions and form two novel zinc-binding motifs. The first and second cysteine clusters (residues 111–116, 138–143, respectively) form two zinc-binding motifs together with the conserved Cys103 and His122, respectively. Both zinc ions interact with helix α5 and stabilize the structure of the C-terminal unique domain.

The Aα subunit of PP2A consists of 15 HEAT repeats, with each repeat containing two antiparallel helices. Overall the PP2A Aα subunit forms a horseshoe shape structure. SV40 ST “rides” on the structural ridge formed by intra-HEAT-repeat loops of PP2A Aα subunit (Figure 1). Previous work has demonstrated that the unique domain of SV40 ST is the essential binding domain for PP2A AC core complex [5]. In our structure the second zinc-binding motif, in particular the L6 loop, forms extensive interactions with the PP2A Aα subunit. In support of our structural observations, in vitro structure-directed mutagenesis studies demonstrate that the interface between the second zinc-binding motif, in particular Pro132 that intercalates between Trp140 and Phe141 of PP2A A subunit, is essential for A-ST interactions (Figure 7). Point mutations of two residues in the ST J domain (R7A and R21A), or mutation of an A subunit residue that forms a hydrogen bond with ST Arg21 (E216A, Figure 3), all abolish the A-ST interaction (Figure 7), confirming the J domain is directly involved in A subunit interaction. In addition, this structure is completely consistent with previous mutagenesis studies of PP2A A subunit [44], since essentially all A subunit mutations that have weaker ST binding activities map to the A-ST interface in our structure.

### Potential Interaction between ST and PP2A C Subunit

Previous work has indicated that a region of the unique domain encompassing the first cysteine cluster is necessary for the binding of ST to PP2A, and its N-terminal flanking region (residues 97–103) are also important for PP2A interactions [35,47]. However, the first zinc-binding motif does not interact with the A subunit of PP2A in the crystal structure. Instead, the crystal structure suggests that the first zinc-binding motif may directly interact with the C subunit near its active site, since the first zinc-binding motif is spatially close to the active site of the PP2A C subunit in the structural superposition of PP2A and A-ST complexes (Figure 5). This hypothesis is supported by structural flexibility of the scaffold A subunit. Our structural comparison indicates that HEAT repeats 10–13 of PP2A Aα subunit have substantial structural flexibility (Figure 6). This flexibility of the Aα subunit may allow for the accommodation of different types of regulatory B subunits or B-like proteins, such as ST, into the AC core enzyme that interact with both A and C subunit.

Consistent with prior reports [23,24], we failed to observe stable direct interaction between purified ST and PP2A C subunit using a glutathione S-transferase (GST) pull-down assay (unpublished data). It is possible that ST directly interacts with the C subunit via the formation of a stable complex between ST and the A subunit. Therefore, ST may form trivalent interactions with the PP2A AC complex—two of them (via the J domain and the second zinc-binding motif) interacting with the A subunit, and the third one (via the first zinc-binding motif) binding to the C subunit. This hypothesis explains why the first zinc-binding motif does not directly interact with PP2A A subunit, yet is required for the interaction with and the inhibition of phosphatase activity of PP2A AC core complex [35]. This model is also supported by the observation that ST fragments containing both J domain and the first but not the second zinc-binding motif interact with PP2A AC complex and inhibit PP2A AC dimer phosphatase activity [29,35]. ST may interfere with substrate binding via its interaction near the active site of the C subunit. Therefore, in addition to competing with the PP2A B subunit for PP2A A subunit binding, ST may directly modulate the phosphatase activity of the AC core complex, which accounts for substantial proportion of PP2A enzyme in the cell. Future work will be needed to understand if and how ST may directly interact with the C subunit.

### The Role of the ST J Domain in the Interaction with PP2A

Prior studies have demonstrated that the unique domain but not the J domain was sufficient for interaction with PP2A AC complex. Although not essential for A-ST interaction, the deletion of the J domain significantly decreased the inhibitory activity of ST on the PP2A AC core dimer [35], suggesting that the J domain enhances the binding of ST to the PP2A A subunit. Consistent with this view, mutation of either Arg7 or Arg21, two residues on the J domain surface involved in PP2A A interaction, disrupts the interaction between ST and A subunit. Alternatively, the J domain may play a role in stabilizing the spatial position of the first zinc-binding motif by allowing its efficient interaction with the C subunit. This interaction may be particularly important in the AC-ST complex formation, because the structural flexibility of HEAT repeats 10–13 of the A subunit may not permit the C subunit to stay in a fixed position and interact with ST efficiently. Indeed, although the unique domain of ST binds to PP2A A, this binding fails to inhibit PP2A AC phopshatase activity [35].

In this regard, we note that the second zinc-binding motif binds to the A subunit primarily through loop–loop interactions and on the concave side of the A subunit structure only, while the J domain interacts with the convex side of A subunit. While the second zinc-binding motif may be the primary docking site, the J domain may fix the relative orientation between ST unique domain and the A subunit, with the N-terminal J and C-terminal unique domains sitting on the convex and concave side of the horseshoe shape, respectively. This orientation stabilization may be important for the first zinc-binding motif to effectively inhibit the phosphatase activity of the PP2A C subunit.

In addition to the inhibition of the phosphatase activity of the PP2A AC dimer, the J domain may also play a role in the oncogenic activity of ST by providing an additional binding site for Hsp70, even when in complex with the PP2A AC complex, as suggested by our crystal structure. The potential simultaneous interaction with PP2A and Hsp70 may couple these two functions of ST. For example, ST may bring PP2A and Hsp70 together to allow for the dephosphorylation of protein(s) bound to Hsp70.

In summary, our structural and biochemical studies reveal the structure of the ST family and define the interaction between ST and the A subunit of PP2A. In addition, our work suggests that ST may directly interact and regulate the activity or substrate specificity of the PP2A catalytic C subunit, and Hsp70 may bind to PP2A-bound ST and thus define PP2A activity and/or substrate specificity. Taken together, our work provides a structural basis for the oncogenic activity of ST and MT antigens in the polyomavirus family. Since ST binds to PP2A Aα in a manner similar to that used by the regulatory B subunits, these findings provide not only new insights into the regulation of PP2A but may also provide a foundation for the development of small molecules that alter the function of PP2A.

## Materials and Methods

### Expression and purification of SV40 ST in complex with PP2A A subunit.

Full-length SV40 ST (strain VA45‐54‐2) and full-length mouse PP2A Aα subunit with tobacco etch virus protease (TEV) cleavage sites were cloned into pGEX4T1 vector (Amersham Biotech, http://www.gelifesciences.com) and pET28a vector (Novagen, http://www.emdbiosciences.com/html/NVG/home.html), respectively, and co-transformed into E. coli strain BL21 (star) (Invitrogen, http://www.invitrogen.com). Mouse and human Aα subunits are identical in protein sequence, except for one residue that is distant from the ST binding site (Ser324 in human, Thr324 in mouse). Coexpression of Aα subunit and SV40 ST was induced by the addition of 0.1 mM IPTG at OD_600_ = 0.6 upon shifting the temperature from 37 °C to 18 °C, and cells were grown for an additional 18 h. Cells were then collected by centrifugation and resuspended with lysis buffer (30 mM Tris-HCl [pH 8.0], 50 mM NaCl, 5 mM β-mercaptoethanol) including protease inhibitors (PMSF, leupeptin, and benzamidine). Resuspended cells were lysed by sonication, and cell debris removed by centrifugation at 26,000 *g* for 1 h. Soluble fractions were filtered with 0.8 μm syringe filters and applied into a Ni-NTA affinity column pre-equilibrated with 30 mM Tris-HCl (pH 8.0), 50 mM NaCl, 5 mM β-mercaptoethanol. Target protein complexes (the Aα subunit with GST-tag and SV40 ST with His-tag) were eluted with elution buffer (30 mM Tris-HCl [pH 8.0], 50 mM NaCl, 300 mM imidazole, 5 mM β-mercaptoethanol) and dialyzed overnight at 4 °C in 30 mM Tris-HCl (pH 8.0), 50 mM NaCl, 5 mM DTT. Dialyzed protein was applied to a GST affinity column to remove free SV40 ST, and on-column cleavage with TEV protease was performed at 4 °C overnight. The flow-through fraction of the GST column was reapplied into the Ni-NTA column to remove cleaved His-tag and TEV protease. The flow-through fraction of the Ni-NTA column was concentrated and applied into a Superdex 200 size-exclusion column (Amersham Biotech) pre-equilibrated with 30 mM Tris-HCl (pH 8.0), 50 mM NaCl, 5 mM DTT. Fractions of the heterodimeric complex of full-length Aα subunit and full-length SV40 ST were pooled and concentrated up to 10 mg/ml and used for the crystallization trial. Crystals of the A-ST complex were obtained at room temperature with the hanging drop vapor diffusion method. After optimizing initial conditions and extensively searching for additives, combined with microseeding, the optimal crystallization condition was 16% PEG 3350, 0.2 M ammonium formate, 30 mM spermine, 6% 6-aminocaproic acid, 10 mM DTT. Crystals were cryoprotected by gradually increasing the PEG 3350 concentration up to 26% and frozen with liquid nitrogen.

### Data collection and structure determination of the PP2A Aα subunit and SV40 ST complex.

Data collection was performed at Advanced Light Source beamline 5.0.1. The best dataset has a resolution of 3.1 Å, with high redundancy. The space group was P1, with the unit cell dimensions a = 94.69 Å, b = 105.50 Å, c = 111.98 Å, α = 115.64°, β = 109.57°, γ = 94.10°. These datasets were integrated and scaled using HKL2000 and SCALEPACK [48]. There were four heterodimers in one asymmetric unit with 54% solvent content. Initial molecular replacement was performed with Phaser [49] by using the Aα subunit structure in the trimeric PP2A complex [25] as the search model. The program Phaser found all four Aα subunits with pseudo D2 symmetry. However, both *R*_free_ and *R*_work_ were over 55% after overall rigid-body refinement, suggesting substantial conformational changes in the Aα subunit. Therefore, rigid body refinement was performed in Refmac5, with every single HEAT repeat as a separate group [50]. After this rigid body refinement, *R*_free_ dropped to about 40%. Since SV40 ST was demonstrated to contain two zinc-binding sites [34] that gave significant anomalous signal at the experimental wavelength (λ = 1.000 Å), the same dataset was scaled as an anomalous dataset using HKL2000. The anomalous difference map, calculated using phases derived from the refined molecular replacement solution, gave the positions of eight zinc sites (for four ST molecules in the asymmetric unit) without any ambiguity. Zinc sites were further refined with Sharp [51], and phases were calculated together with the partial model of the Aα subunit. The calculated map showed clear density of SV40 ST after the density modification with DM. Since the conformation of each four Aα subunit was different, noncrystallographic symmetry averaging was helpful only to build SV40 ST. Further model building was done with Xtalview [52] and COOT [53]. TLS refinement was performed using Refmac5 in the CCP4 suite [50]. Because of the conformational variations in the A subunit, noncrystallographic symmetry averaging was not applied during refinement. The final model has an *R*_free_ of 30.5%, and an *R*_work_ of 24.4%. Zinc sites are confirmed by anomalous difference map. None of the nonglycine residues are in the disallowed region of the Ramanchandran plot. The final structural models of ST contain residues 1–44, 48–78, 85–172 (chain e); 1–40, 49–74, 87–172 (chain f); 1–41, 51–70, 92–172 (chain g); and 1–41, 51–73, 94–172 (chain h).

### In vitro binding assay.

GST-tagged ST and Aα mutants were generated using the QuickChange Site-Directed Mutagenesis Kit (Stratagene) and expressed in E. coli strain BL21 (star). Wild-type Aα and Aα mutants were isolated with glutathione-sepharose (Amersham Biosciences) GST-tags were removed from wild-type ST and ST mutants and equal amounts of wild-type ST or ST mutants were added to Aα-glutathione-sepharose precipitates. Binding assays were performed in 30 mM Tris (pH 8.0), 50 mM NaCl, 5 mM DTT, 0.2% NP-40 buffer for 4 h. The beads were washed five times, and the proteins were eluted with reduced glutathione, followed by SDS-PAGE and immunoblotting. For immunoblotting, we used affinity-purified polyclonal antibodies against SV40 ST [16] and monoclonal antibodies (clone 6F9) against Aα (Abcam, http://www.abcam.com).

## Supporting Information

Figure S1 Structural Comparison of Individual HEAT Repeats of the PP2A A Subunit Identical individual HEAT repeats of the PP2A A subunit (repeats 10–13) were superimposed to visualize the conformational changes of residues in these HEAT repeats. Seven structures were used in the superposition: the four A-ST complexes in the asymmetric unit (this work), the A subunit alone (Protein Data Bank [PDB] code: 1B3U), the AC dimer (PDB code: 2IE3), and the AB′C trimer (PDB code: 2IAE). It appears that conformational flexibility of HEAT repeats 10–13 is an accumulative effect of numerous residues.
(5.0 MB TIF)

Figure S2 Structural Comparison of Inter-repeat Orientations of the PP2A A Subunit The seven different A subunit structures, same as these used in Figure S1, were superimposed in HEAT repeats 10–11, 11–12, and 12–13. Only the N-terminal repeat (shown in front in the top panel) was used for the alignment to visualize the inter-repeat orientational changes of the following repeat. Major inter-repeat orientational changes in the PP2A A subunit were observed between HEAT repeats 10–11 and 12–13. The inter-repeat change between repeats 11 and 12 is relatively small compared with these of repeats 10–11 and 12–13.
(3.9 MB TIF)

### Accession Numbers

Coordinates and structural factors have been deposited in the Protein Data Bank (PDB, http://www.rcsb.org/pdb) with the accession code 2PF4.

## Abbreviations

GST: glutathione S-transferase
HEAT: huntingtin, elongation factor 3, A subunit of protein phosphatase 2A, and TOR (target of rapamycin)
Hsp70: heat shock protein 70
LT: large T antigen
MT: middle T antigen
PDB: Protein Data Bank
PP2A: protein phosphatase 2A
RMSD: root mean square deviation
ST: small t antigen
SV40: simian virus 40
TEV: tobacco etch virus protease

## References

[pbio-0050202-b001] Barbanti-Brodano G, Sabbioni S, Martini F, Negrini M, Corallini A (2004). Simian virus 40 infection in humans and association with human diseases: Results and hypotheses. Virology.

[pbio-0050202-b002] Skoczylas C, Fahrbach KM, Rundell K (2004). Cellular targets of the SV40 small-t antigen in human cell transformation. Cell Cycle.

[pbio-0050202-b003] Rundell K, Parakati R (2001). The role of the SV40 ST antigen in cell growth promotion and transformation. Semin Cancer Biol.

[pbio-0050202-b004] Yu J, Boyapati A, Rundell K (2001). Critical role for SV40 small-t antigen in human cell transformation. Virology.

[pbio-0050202-b005] Hahn WC, Dessain SK, Brooks MW, King JE, Elenbaas B (2002). Enumeration of the simian virus 40 early region elements necessary for human cell transformation. Mol Cell Biol.

[pbio-0050202-b006] Rangarajan A, Hong SJ, Gifford A, Weinberg RA (2004). Species- and cell type-specific requirements for cellular transformation. Cancer Cell.

[pbio-0050202-b007] Sontag E (2001). Protein phosphatase 2A: The Trojan horse of cellular signaling. Cell Signal.

[pbio-0050202-b008] Janssens V, Goris J (2001). Protein phosphatase 2A: A highly regulated family of serine/threonine phosphatases implicated in cell growth and signalling. Biochem J.

[pbio-0050202-b009] Goldberg Y (1999). Protein phosphatase 2A: Who shall regulate the regulator?. Biochem Pharmacol.

[pbio-0050202-b010] Virshup DM (2000). Protein phosphatase 2A: A panoply of enzymes. Curr Opin Cell Biol.

[pbio-0050202-b011] Ruediger R, Hentz M, Fait J, Mumby M, Walter G (1994). Molecular model of the A subunit of protein phosphatase 2A: Interaction with other subunits and tumor antigens. J Virol.

[pbio-0050202-b012] Ruediger R, Pham HT, Walter G (2001). Disruption of protein phosphatase 2A subunit interaction in human cancers with mutations in the A alpha subunit gene. Oncogene.

[pbio-0050202-b013] Ito A, Kataoka TR, Watanabe M, Nishiyama K, Mazaki Y (2000). A truncated isoform of the PP2A B56 subunit promotes cell motility through paxillin phosphorylation. EMBO J.

[pbio-0050202-b014] MacKintosh C, MacKintosh RW (1994). Inhibitors of protein kinases and phosphatases. Trends Biochem Sci.

[pbio-0050202-b015] Chen W, Hahn WC (2003). SV40 early region oncoproteins and human cell transformation. Histol Histopathol.

[pbio-0050202-b016] Chen W, Possemato R, Campbell KT, Plattner CA, Pallas DC (2004). Identification of specific PP2A complexes involved in human cell transformation. Cancer Cell.

[pbio-0050202-b017] Wang SS, Esplin ED, Li JL, Huang L, Gazdar A (1998). Alterations of the PPP2R1B gene in human lung and colon cancer. Science.

[pbio-0050202-b018] Ruediger R, Pham HT, Walter G (2001). Alterations in protein phosphatase 2A subunit interaction in human carcinomas of the lung and colon with mutations in the A beta subunit gene. Oncogene.

[pbio-0050202-b019] Takagi Y, Futamura M, Yamaguchi K, Aoki S, Takahashi T (2000). Alterations of the PPP2R1B gene located at 11q23 in human colorectal cancers. Gut.

[pbio-0050202-b020] Tamaki M, Goi T, Hirono Y, Katayama K, Yamaguchi A (2004). PPP2R1B gene alterations inhibit interaction of PP2A-Abeta and PP2A-C proteins in colorectal cancers. Oncol Rep.

[pbio-0050202-b021] Chen W, Arroyo JD, Timmons JC, Possemato R, Hahn WC (2005). Cancer-associated PP2A alpha subunits induce functional haploinsufficiency and tumorigenicity. Cancer Res.

[pbio-0050202-b022] Sablina AA, Chen W, Arroyo JD, Corral L, Hector M (2007). The tumor suppressor PP2A A beta regulates the RalA GTPase. Cell.

[pbio-0050202-b023] Yang SI, Lickteig RL, Estes R, Rundell K, Walter G (1991). Control of protein phosphatase 2A by simian virus 40 small-t antigen. Mol Cell Biol.

[pbio-0050202-b024] Ruediger R, Roeckel D, Fait J, Bergqvist A, Magnusson G (1992). Identification of binding sites on the regulatory A subunit of protein phosphatase 2A for the catalytic C subunit and for tumor antigens of simian virus 40 and polyomavirus. Mol Cell Biol.

[pbio-0050202-b025] Cho US, Xu W (2007). Crystal structure of a protein phosphatase 2A heterotrimeric holoenzyme. Nature.

[pbio-0050202-b026] Xu Y, Xing Y, Chen Y, Chao Y, Lin Z (2006). Structure of the protein phosphatase 2A holoenzyme. Cell.

[pbio-0050202-b027] Pallas DC, Shahrik LK, Martin BL, Jaspers S, Miller TB (1990). Polyoma small and middle T antigens and SV40 small t antigen form stable complexes with protein phosphatase 2A. Cell.

[pbio-0050202-b028] Kamibayashi C, Estes R, Lickteig RL, Yang SI, Craft C (1994). Comparison of heterotrimeric protein phosphatase 2A containing different B subunits. J Biol Chem.

[pbio-0050202-b029] Sontag E, Fedorov S, Kamibayashi C, Robbins D, Cobb M (1993). The interaction of SV40 small tumor antigen with protein phosphatase 2A stimulates the map kinase pathway and induces cell proliferation. Cell.

[pbio-0050202-b030] Scheidtmann KH, Mumby MC, Rundell K, Walter G (1991). Dephosphorylation of simian virus 40 large-T antigen and p53 protein by protein phosphatase 2A: Inhibition by small-t antigen. Mol Cell Biol.

[pbio-0050202-b031] Boyapati A, Wilson M, Yu J, Rundell K (2003). SV40 17KT antigen complements dnaj mutations in large T antigen to restore transformation of primary human fibroblasts. Virology.

[pbio-0050202-b032] Arroyo JD, Hahn WC (2005). Involvement of PP2A in viral and cellular transformation. Oncogene.

[pbio-0050202-b033] Goswami R, Turk B, Enderle K, Howe A, Rundell K (1992). Effect of zinc ions on the biochemical behavior of simian virus 40 small-t antigen expressed in bacteria. J Virol.

[pbio-0050202-b034] Turk B, Porras A, Mumby MC, Rundell K (1993). Simian virus 40 small-t antigen binds two zinc ions. J Virol.

[pbio-0050202-b035] Mateer SC, Fedorov SA, Mumby MC (1998). Identification of structural elements involved in the interaction of simian virus 40 small tumor antigen with protein phosphatase 2A. J Biol Chem.

[pbio-0050202-b036] Walsh P, Bursac D, Law YC, Cyr D, Lithgow T (2004). The J-protein family: Modulating protein assembly, disassembly and translocation. EMBO Rep.

[pbio-0050202-b037] Cheetham ME, Caplan AJ (1998). Structure, function and evolution of DnaJ: Conservation and adaptation of chaperone function. Cell Stress Chaperones.

[pbio-0050202-b038] Greene MK, Maskos K, Landry SJ (1998). Role of the J-domain in the cooperation of Hsp40 with Hsp70. Proc Natl Acad Sci U S A.

[pbio-0050202-b039] Suh WC, Burkholder WF, Lu CZ, Zhao X, Gottesman ME (1998). Interaction of the Hsp70 molecular chaperone, DnaK, with its cochaperone DnaJ. Proc Natl Acad Sci U S A.

[pbio-0050202-b040] Whalen KA, de Jesus R, Kean JA, Schaffhausen BS (2005). Genetic analysis of the polyomavirus DnaJ domain. J Virol.

[pbio-0050202-b041] Kim HY, Ahn BY, Cho Y (2001). Structural basis for the inactivation of retinoblastoma tumor suppressor by SV40 large T antigen. EMBO J.

[pbio-0050202-b042] Berjanskii MV, Riley MI, Xie A, Semenchenko V, Folk WR (2000). NMR structure of the N-terminal J domain of murine polyomavirus T antigens. Implications for DnaJ-like domains and for mutations of T antigens. J Biol Chem.

[pbio-0050202-b043] Vallee BL, Coleman JE, Auld DS (1991). Zinc fingers, zinc clusters, and zinc twists in DNA-binding protein domains. Proc Natl Acad Sci U S A.

[pbio-0050202-b044] Ruediger R, Fields K, Walter G (1999). Binding specificity of protein phosphatase 2A core enzyme for regulatory B subunits and T antigens. J Virol.

[pbio-0050202-b045] Groves MR, Hanlon N, Turowski P, Hemmings BA, Barford D (1999). The structure of the protein phosphatase 2A PR65/A subunit reveals the conformation of its 15 tandemly repeated HEAT motifs. Cell.

[pbio-0050202-b046] Xing Y, Xu Y, Chen Y, Jeffrey PD, Chao Y (2006). Structure of protein phosphatase 2A core enzyme bound to tumor-inducing toxins. Cell.

[pbio-0050202-b047] Mungre S, Enderle K, Turk B, Porras A, Wu YQ (1994). Mutations which affect the inhibition of protein phosphatase 2A by simian virus 40 small-t antigen in vitro decrease viral transformation. J Virol.

[pbio-0050202-b048] Otwinowski Z, Minor W (1996). Processing of X-ray diffraction data collected in oscillation mode. Methods Enzymol.

[pbio-0050202-b049] Read RJ (2001). Pushing the boundaries of molecular replacement with maximum likelihood. Acta Crystallogr D Biol Crystallogr.

[pbio-0050202-b050] Murshudov GN, Vagin AA, Lebedev A, Wilson KS, Dodson EJ (1999). Efficient anisotropic refinement of macromolecular structures using FFT. Acta Crystallogr D Biol Crystallogr.

[pbio-0050202-b051] De La Fortelle E, Bricogne G (1997). Maximum-likelihood heavy-atom parameter refinement for multiple isomorphous replacement and multiwavelength anomalous diffraction methods. Methods Enzymol.

[pbio-0050202-b052] McRee DE (1999). XtalView/Xfit—A versatile program for manipulating atomic coordinates and electron density. J Struct Biol.

[pbio-0050202-b053] Emsley P, Cowtan K (2004). Coot: Model-building tools for molecular graphics. Acta Crystallogr D Biol Crystallogr.

