# Supplementary figures and images for: Structural Basis of PP2A Inhibition by Small t Antigen
Source: PLoS Biol. 2007 Jul 3;5(8):e202. doi: 10.1371/journal.pbio.0050202 (PMC1945078; doi:10.1371/journal.pbio.0050202)

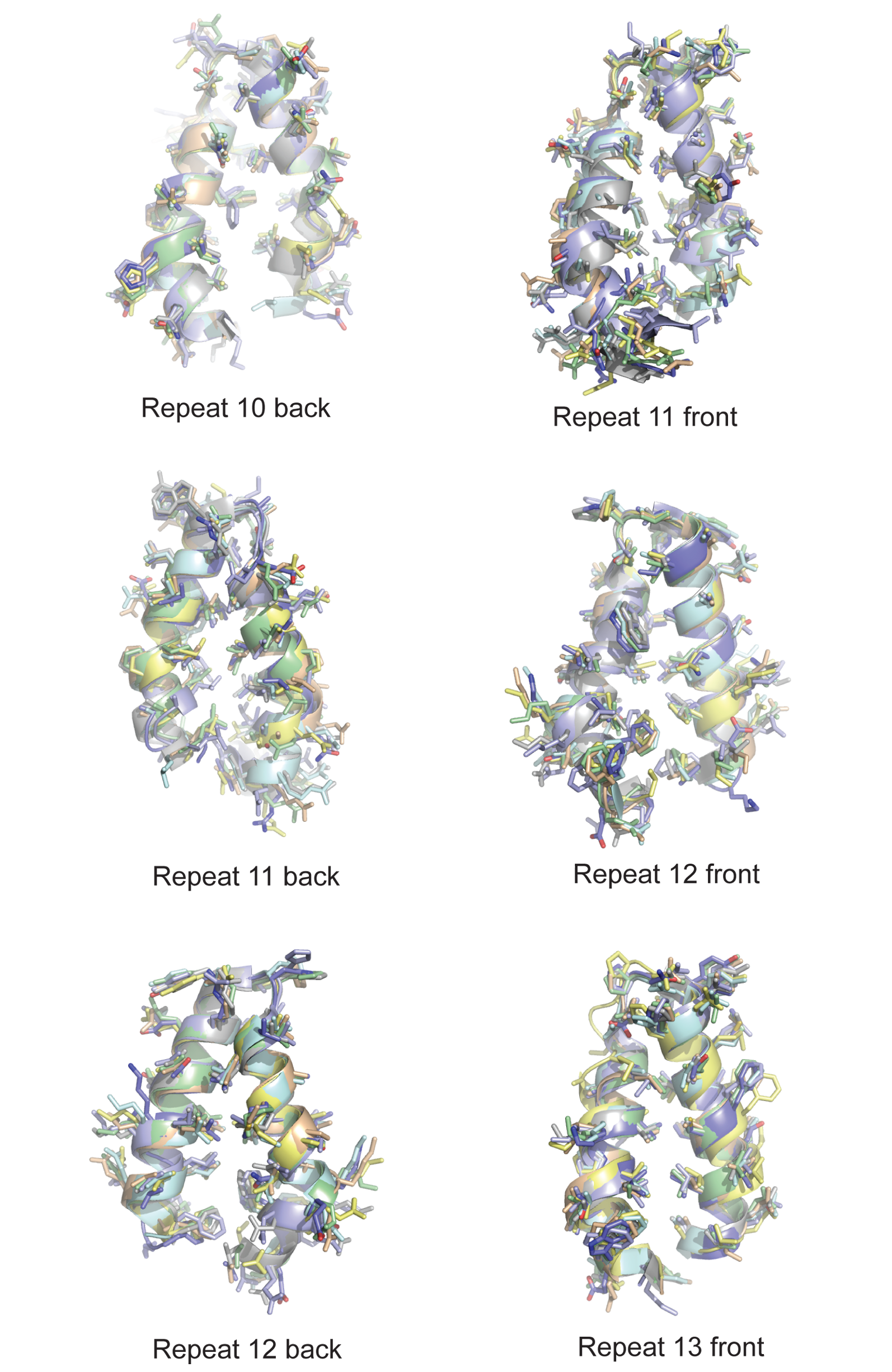

Supplement: Figure S1 — Identical individual HEAT repeats of the PP2A A subunit (repeats 10–13) were superimposed to visualize the conformational changes of residues in these HEAT repeats. Seven structures were used in the superposition: the four A-ST complexes in the asymmetric unit (this work), the A subunit alone (Protein Data Bank [PDB] code: 1B3U), the AC dimer (PDB code: 2IE3), and the AB′C trimer (PDB code: 2IAE). It appears that conformational flexibility of HEAT repeats 10–13 is an accumulative effect of numerous residues. (5.0 MB TIF) [file pbio.0050202.sg001.tif]

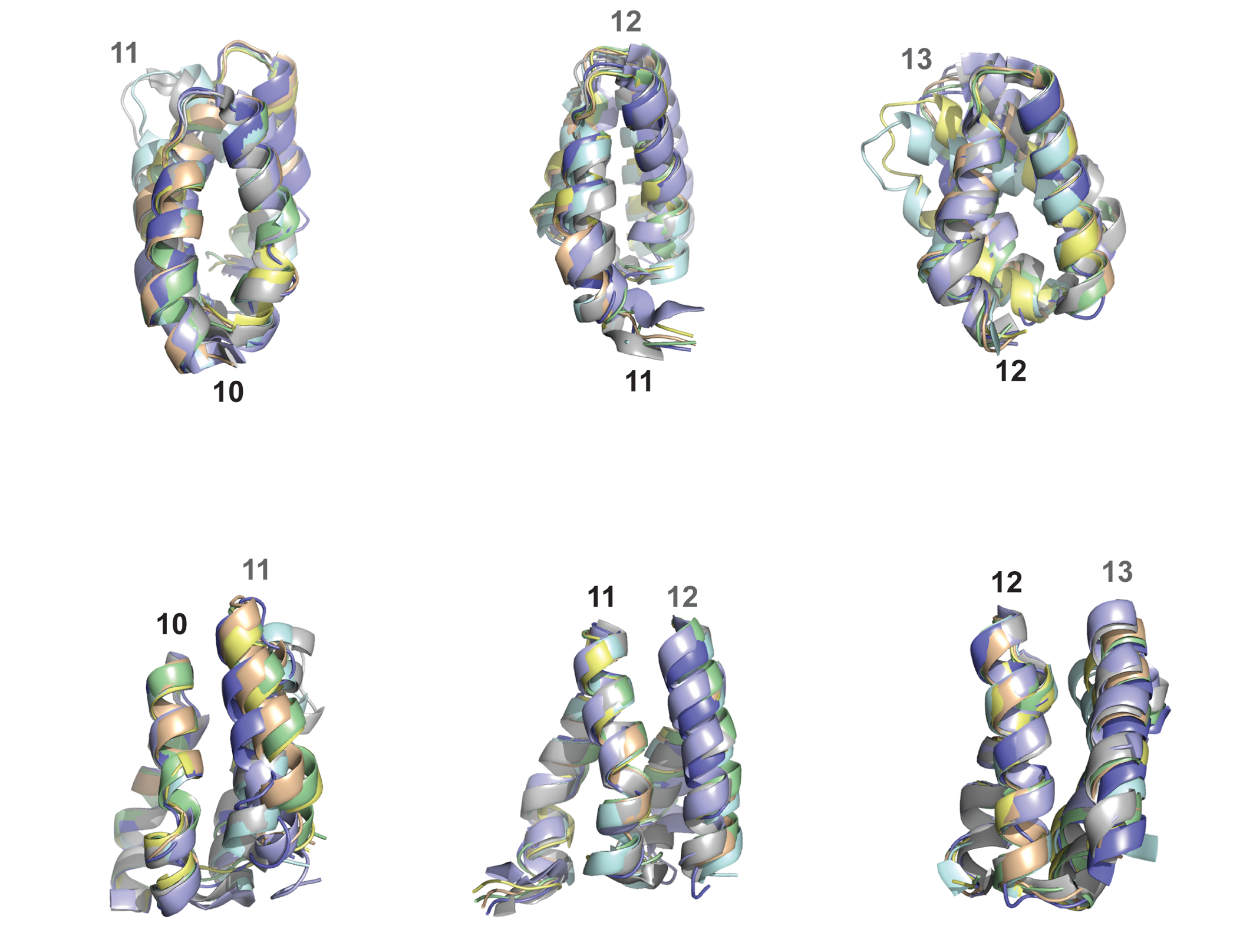

Supplement: Figure S2 — The seven different A subunit structures, same as these used in Figure S1, were superimposed in HEAT repeats 10–11, 11–12, and 12–13. Only the N-terminal repeat (shown in front in the top panel) was used for the alignment to visualize the inter-repeat orientational changes of the following repeat. Major inter-repeat orientational changes in the PP2A A subunit were observed between HEAT repeats 10–11 and 12–13. The inter-repeat change between repeats 11 and 12 is relatively small compared with these of repeats 10–11 and 12–13. (3.9 MB TIF) [file pbio.0050202.sg002.tif]
